# Enhancement of Ti(C,N)-Based Cermets by Aluminum Nitride: Binder Phase Strengthening and Microstructural Refinement

**DOI:** 10.3390/ma19122441

**Published:** 2026-06-07

**Authors:** Lili Ma, Yiping Xu, Yulin Lin, Binbin Wei, Houan Zhang

**Affiliations:** 1 Fujian Province Key Laboratory of Functional Materials and Applications, Xiamen University of Technology, Xiamen 361024, China; xyp18922482782@163.com (Y.X.); bbwei@xmut.edu.cn (B.W.); 2 School of Materials Science and Engineering, Xiamen University of Technology, Xiamen 361024, China; 3 Zhangzhou Hyun Cutting Tools Co., Ltd., Zhangzhou 363601, China; linyulin1979@163.com

**Keywords:** Ti(C,N)-based cermets, aluminum nitride, microstructure, mechanical properties, binder phase

## Abstract

This study systematically investigated the influence of aluminum nitride (AlN) addition on the microstructure and mechanical properties of Ti(C,N)-based cermets with low Ni/Co and high N contents. The results demonstrate that AlN addition does not alter the phase composition of Ti(C,N)-based cermets, and their microstructure retains the characteristic core–rim morphology. The dissolution of Al atoms from AlN effectively suppressed abnormal growth of Ti(C,N) grains and enhanced the mechanical stability of the binder. Densification and comprehensive mechanical properties strongly depended on the AlN content. Both parameters initially improved with the addition of AlN, peaked at an optimal content of 1.0 wt.%, and then declined upon further addition due to the formation of micropores and brittle agglomerates of Al_2_O_3_. At the optimal AlN content of 1.0 wt.%, the cermets demonstrated peak performance: a Vickers hardness of 1676 HV30, a transverse rupture strength of 1486 MPa, and a fracture toughness of 8.8 MPa·m^1/2^. The results elucidate the dual role of AlN as both a microstructural refiner and a precursor for in situ strengthening of the binder phase.

## 1. Introduction

Ti(C,N)-based cermets have attracted considerable interest because of their high red hardness, good wear resistance, abundant raw material availability, and notable high-temperature oxidation resistance [1,2,3,4]. Owing to these advantages, they can replace conventional WC-Co cemented carbides in cutting tool applications [5]. The cermet system develops its characteristic core–rim–binder phase architecture through dissolution–reprecipitation processes that occur during liquid-phase sintering [6,7]. Prior studies indicate that adding WC and Mo_2_C improves the wettability between the binder and hard phases, whereas the combined introduction of Mo_2_C and TaC enhances the overall mechanical performance. However, compared with WC-Co cemented carbides, Ti(C,N)-based cermets still possess lower fracture toughness and flexural strength, which remains a major barrier to their wider industrial adoption [8,9].

Aluminum nitride (AlN) possesses adequate high-temperature oxidation resistance and stability [10]. In a study by Lin et al., the addition of AlN to WC-Co cemented carbides inhibited the growth of WC grains, thereby enhancing their mechanical properties through the mechanisms of grain refinement strengthening and dispersion strengthening [11]. Additionally, some researchers have suggested that the incorporation of nitrogen from AlN into Ti(C,N)-based cermets may be beneficial for improving their toughness. Consequently, they introduced AlN powder into TiCN-based cermets and discovered that the aluminum introduced via AlN addition reduced the thickness of the ceramic phase rim. This reduction increases nitrogen activity, thereby refining the grains and further enhancing the hardness and transverse rupture strength of the cermets [12]. Additionally, other researchers have reported that the oxidation resistance of AlN-reinforced Ti(C,N)-based cermets is enhanced [13]. However, most of the above studies focused on Ti(C,N)-based cermets with relatively low nitrogen content (e.g., TiC_0.6_N_0.4_) and a high mass fraction of the binder phase (e.g., >20 wt.%). The increasing utilization of Ti(C,N)-based cermets—characterized by high nitrogen content and a low binder phase mass fraction—calls for a systematic investigation into the strengthening mechanism of aluminum nitride (AlN) in such materials.

It is well established that increasing the nitrogen content in Ti(C,N) elevates its nitrogen dissociation partial pressure during sintering. In high-nitrogen systems, how does nitrogen released from AlN decomposition affect densification behavior—particularly relative to the low-nitrogen reference systems documented in the literature [12]? Moreover, although prior studies have confirmed that Al atoms originating from AlN dissolution strengthen the binder phase upon uniform dispersion therein, the consequences for compositions with low binder phase content remain poorly understood. Specifically, is the optimal AlN addition level correspondingly reduced in such low-binder-phase systems? Guided by our group’s prior experience, a Ti(C,N)-based cermet featuring both high nitrogen content (using TiC_0.5_N_0.5_ as the raw material) and a low binder phase fraction (16 wt.%) was selected as the model system [14]. This study systematically investigates the effect of AlN addition (0–3.0 wt.%) on the microstructure and mechanical properties of high-nitrogen (TiC_0.5_N_0.5_), low-binder (16 wt.%) Ti(C,N)-based cermets, and elucidates the role of AlN both as a nitrogen source influencing pore evolution and as a solute strengthening agent in the binder phase.

## 2. Experimental Procedures

### 2.1. Cermet Preparation

**Figure 1:**
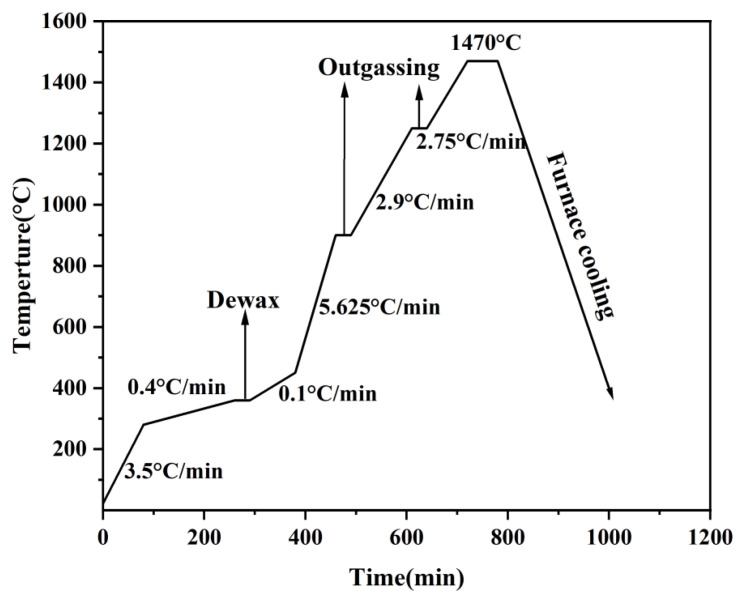
Sintering procedure for Ti(C,N)-based cermets under vacuum.

To prepare Ti(C,N)-based cermets with different AlN contents, commercial Ti(C_0.5_N_0.5_), WC, Mo_2_C, NbC, Ni, Co, and carbon powders were used. Samples containing 0 wt.%, 1.0 wt.%, 2.0 wt.%, and 3.0 wt.% AlN were synthesized and designated A0, A1, A2, and A3, respectively. The AlN powder was stored in a sealed bag under vacuum. Table 1 summarizes the particle size distribution, suppliers, and purity of the starting powder materials used in this study, while Table 2 lists the cermet compositions with different AlN additions. A total of 4.0 wt.% PEG4000 and PEG1500 were added as forming agents or lubricants. The weighed raw powders were ball-milled with WC-Co balls in deionized water (in an air atmosphere inside the jar) using a tumbling ball mill at a rotational speed of 130 rpm, with a ball-to-powder mass ratio of 10:1. Milling was conducted for 60 h. The resulting slurry was filtered through a 250 mesh screen to maintain uniformity. The filtered slurry was then spray-dried at 110 °C to obtain granulated mixed powders. The mixed powder was compacted under a uniaxial pressure of 250 MPa into standard specimens with dimensions of 25.5 mm × 8.5 mm × 7 mm [14]. Finally, the as-pressed green compacts were subjected to vacuum sintering in a vacuum furnace using the thermal cycle depicted in Figure 1, yielding the final sintered cermet samples.

### 2.2. Mechanical Property Characterization

The hardness, transverse rupture strength (TRS), and fracture toughness (K_IC_) were systematically characterized for all the sintered cermets. For hardness testing, one representative sample from each composition was ground and polished following a standardized metallographic procedure: sequential grinding with 150-, 400-, and 800-mesh diamond disks, followed by final polishing using a 1 μm diamond suspension on a synthetic cloth. The samples were then ultrasonically cleaned in anhydrous ethanol for 10 min to remove residual abrasives and organic contaminants prior to indentation. Vickers hardness (HV30) was measured under a 30 kgf load (294.2 N) with a dwell time of 15 s using a calibrated microhardness tester (430SVD; ITW Test & Measurement (Shanghai) Co., Ltd., Shanghai, China); five indentations per sample were made, and the average value was reported. TRS was determined via three-point bending tests conducted on a universal testing machine (CMT5105, SANS, China) in accordance with ISO 3327:2016 [15], employing a support span of 15 mm, a crosshead displacement rate of 0.5 mm·min^–1,^ and a minimum of five specimens per composition. Bulk density was measured by Archimedes’ principle using distilled water as the immersion medium, and the theoretical density was calculated from the rule of mixtures based on the constituent phase density. The fracture toughness (K_IC_) was estimated from the Palmqvist crack length and HV30 indentation data by applying the empirical relationship of Anstis et al. [16], with the indentation cracks examined via scanning electron microscopy (SEM):

(1) $$K_{\mathrm{IC}}\;=\;0.15\sqrt{\frac{{\mathrm{HV}}_{30}}{\sum_{i=1}^{4}L_{i}}}$$

where HV30 denotes the Vickers hardness (in kgf·mm^−2^) and L (in mm) represents the length of each Palmqvist crack measured from the indentation corner.

### 2.3. Microstructure Characterization

Microstructural characterization and phase identification were performed using scanning electron microscopy (SEM, Sigma 500, Zeiss, Jena, Germany) equipped with energy-dispersive X-ray spectroscopy (EDS) and X-ray diffraction (XRD, SmartLab-3Kw, Rigaku, Tokyo, Japan) with Cu Kα radiation (λ = 1.5406 Å). SEM imaging was conducted at an accelerating voltage of 15 kV and a working distance of 8 mm to determine the grain morphology, interfacial features, and porosity distribution. EDS elemental mapping and point-by-point analysis were conducted to quantitatively resolve compositional differences between the core and shell regions and to determine the spatial distribution of Al. XRD patterns were collected over a 2θ range of 20–120° at a 0.02°/s step rate. MDI JADE software (version 6.5; Materials Data Ltd., Livermore, CA, USA) was used for numerical analysis of the XRD patterns. The detailed microstructural features were further examined using TEM and high-resolution TEM (HRTEM) with a Talos F200X G2 system (Thermo Scientific, Waltham, MA, USA) operating at 200 kV.

## 3. Results and Discussion

### 3.1. Phase Composition

**Figure 2:**
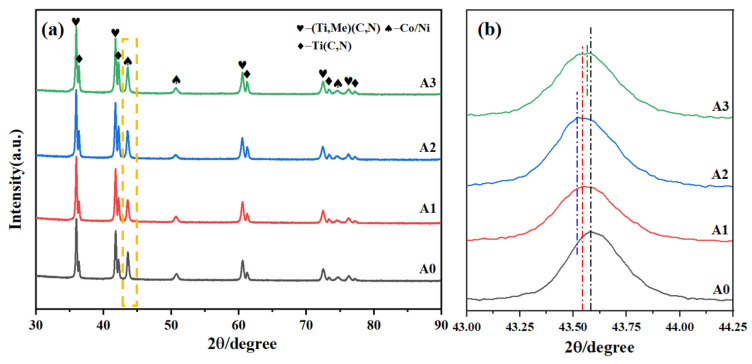
The XRD patterns of the cermet samples with different AlN contents: (**a**) full spectrum; (**b**) magnified view of the 42.5–43.25° region (The dotted lines indicate the positions of the peaks).

The XRD patterns of the sintered cermets containing different amounts of AlN are shown in Figure 2. As revealed in Figure 2a, all four samples exhibit characteristic diffraction peaks attributable to (Ti,Me)(C,N) (Me: W, Mo, Nb heavy element), Ti(C,N), and the Co/Ni binder phase, whereas no diffraction peaks corresponding to raw material components such as WC, Mo_2_C, NbC, and AlN were detected. This indicates that these raw materials either dissolved in the binder phase during sintering or contributed to the formation of the (Ti,Me)(C,N) solid solution phase [14,17]. In addition, no deleterious secondary phases—including free graphite and brittle η-phase compounds—were detected.

The relative intensities of the Ti(C,N) and solid-solution phases were determined from the integrated areas of their respective dominant diffraction peaks—the (200) reflection for Ti(C,N) and the (111) reflection for the solid-solution phase. Compared with sample A0, AlN addition progressively enhances the relative XRD peak intensity of the Ti(C,N) phase, reaching a maximum at 2.0 wt.% AlN (sample A2)-as quantified by the increase in the intensity ratio of the Ti(C,N) (200) peak to the solid-solution (111) peak-from 0.34 in A0 to 0.43 in A2. Beyond this composition, further AlN addition results in negligible change in the Ti(C,N) peak intensity. This observation is likely attributable to the suppression of Ti(C,N) dissolution–precipitation by AlN during the sintering process [12,14]. Furthermore, AlN addition induced a measurable shift in the XRD peak positions of the Co/Ni binder phase, as shown in Figure 2a. To resolve this shift more clearly, the diffraction peak within the range of 42.5–43.25° in Figure 2a, which corresponds to the strongest peak of the Co/Ni phase, was magnified and presented in Figure 2b.

In Figure 2b, the differently colored dotted lines mark the diffraction peak positions of the Co/Ni binder phase in each sample. As the AlN content increases from 0 to 2.0 wt.%, the binder phase peak shifts progressively to lower 2θ angles—indicating lattice expansion due to Al coming from AlN dissolution into the binder phase during sintering [14,18]. A pronounced shift occurs at 1.0 wt.% AlN (sample A1) relative to the AlN-free reference (A0); further increasing AlN to 2.0 wt.% (A2) yields only a marginal additional shift, suggesting near-saturation of Al solubility. In contrast, at 3.0 wt.% AlN (A3), the peak reverses direction and shifts toward higher 2θ angles—nearly recovering the position observed in A0. This phenomenon can be explained by the following mechanism. At low AlN addition levels (≤2.0 wt.%), Al dissolves into the binder phase, causing lattice expansion and consequently a downward shift (to lower 2θ angles) of the diffraction peaks. When the AlN addition becomes excessive (3.0 wt.%), the Al concentration in the binder exceeds its solid solubility limit. This reversal, corroborated quantitatively by the concurrent reduction in lattice parameters listed in Table 3, unambiguously indicates that the Al concentration in A3 exceeds the equilibrium solubility limit in the Co/Ni binder phase. Consequently, Al becomes supersaturated and precipitates as brittle secondary phases—predominantly the thermodynamically stable Al_2_O_3_ (ΔG°f = −1582 kJ/mol)—through reactions with residual oxygen in the system [19]. The preferential formation of Al_2_O_3_ depletes dissolved Al from the binder matrix, thereby counteracting lattice expansion and restoring peak positions toward those of sample A0. However, no diffraction peaks corresponding to AlN or Al_2_O_3_ were detected in the XRD pattern of sample A3. This is expected because conventional XRD has a detection limit of approximately 2–5 vol.% for secondary phases; thus, a small amount of precipitated phase below this threshold cannot be resolved.

### 3.2. Microstructure of the Cermets

**Figure 3:**
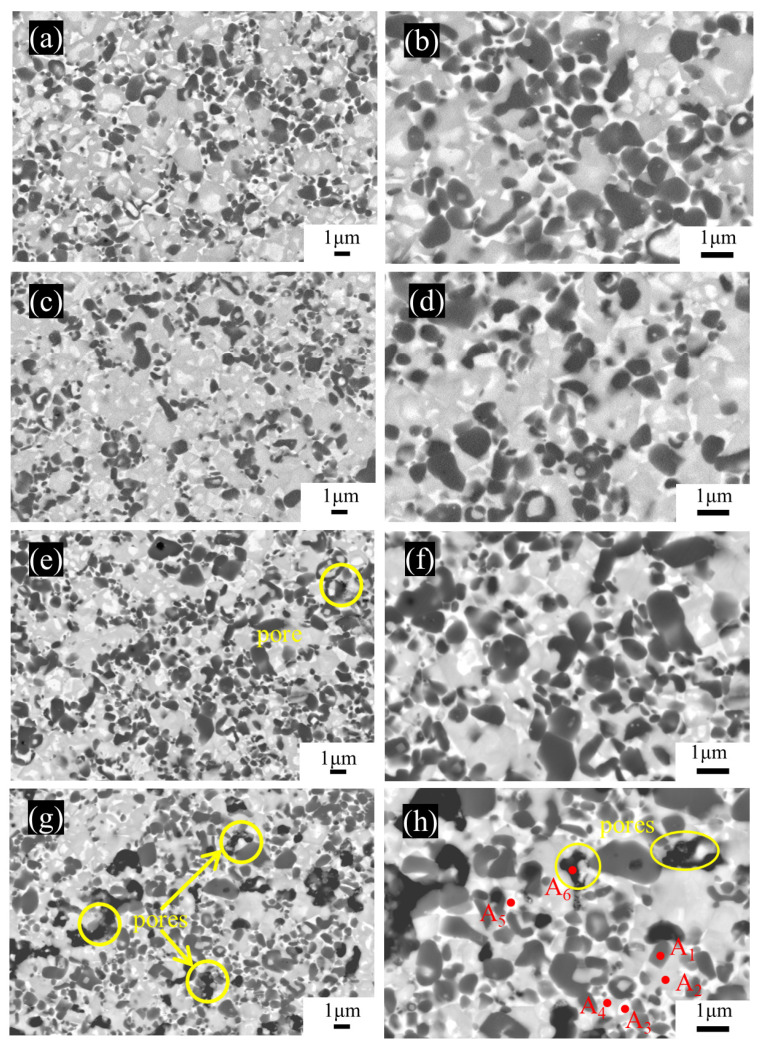
The SEM images of the cermets with varying AlN contents: (**a**,**b**) A0; (**c**,**d**) A1; (**e**,**f**) A2; (**g**,**h**) A3. Points A1–A6 in (**h**) denote the EDS spectrum acquisition spots.

**Figure 4:**
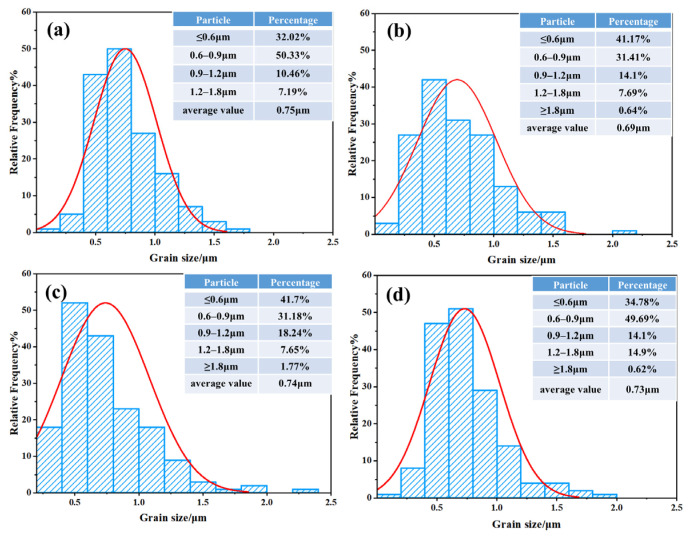
Particle distribution and average grain sizes of the black cores in all four cermet samples. The blue bars represent the frequency distribution of particle sizes, and the red curve indicates the normal distribution fit: (**a**) A0; (**b**) A1; (**c**) A2; (**d**) A3.

Figure 3 presents the backscattered electron (BSE) mode SEM images of cermets with varying AlN contents. The size distribution of the black hard core was quantitatively analyzed using image analysis (ImageJ 1.54f) software and is summarized in Figure 4. As revealed in Figure 3, all cermets display a characteristic core–rim microstructure, consisting of two distinct variants: black core/gray rim and white core/gray rim configurations [14].

In sample A0—prepared without AlN addition (Figure 4a,b)—the black cores exhibit agglomeration and a broad size distribution. With increasing AlN content, the black core size gradually decreases, and its spatial distribution becomes more homogeneous (Figure 3c,d). Quantitative image analysis (Figure 4) confirms that AlN addition systematically reduces the mean black core diameter while concurrently increasing the volume fraction of cores smaller than 0.6 μm—from 32.03% in A0 to 41.7% in A2. The minimum average black core size (0.69 μm) is achieved at 1.0 wt.% AlN. This reduction is attributable to the suppressive effect of AlN on the dissolution–precipitation behavior of the Ti(C,N) phase, as confirmed by XRD analysis—indicating that AlN addition effectively suppresses grain growth [18]. According to the Hall–Petch relationship, grain refinement contributes directly to the improvement of hardness and strength.

However, when the AlN content is increased to 2.0 wt.%, micropores are observed in SEM images of sample A2 (Figure 3e,f). At 3.0 wt.% AlN, both the number and size of the pores increase markedly in sample A3 (Figure 3g,h). Qi Kaifeng et al. investigated the effect of AlN addition on the microstructure of low-nitrogen cermets based on Ti(C,N) (C:N molar ratio = 7:3); they also observed micropore defects in samples containing 5 wt.% AlN and this defects formation was attributed to N_2_ evolution resulting from denitrogenation of Ti(C,N) during liquid-phase sintering [18].

In contrast, the present high-nitrogen system—based on Ti(C,N) with a C:N molar ratio of 5:5—exhibits a relatively higher intrinsic tendency for denitrogenation [3]. Furthermore, nitrogen released from AlN decomposition (via 2AlN → 2Al + N_2_) [19] synergistically accelerates Ti(C,N) denitrogenation during high-temperature processing. As a result, micropore defects emerge even at the low AlN addition level of 2.0 wt.% (sample A2).

It has been established that Al liberated from AlN decomposition readily dissolves into the Co/Ni binder phase [12], promoting the formation of (Ni,Co)_3_Al [13,18,20] intermetallic compounds. Such Al dissolution and associated in situ reactions suppress excessive dissolution–precipitation of Ti(C,N) and second-type carbides in the binder phase. Furthermore, due to the decomposition and dissolution of Al occurring at elevated temperatures [12,14], they facilitate surface-selective precipitation and encapsulation of Ti and other second-type carbides as (Ti,Me)(C,N) solid-solution shells on the hard phases—thereby enabling synergistic regulation of uniform grain growth (Figure 3 and Figure 4).

Notably, the solubility of Al in the binder phase is inherently limited. Excessive addition of AlN leads to Al supersaturation in the binder, prompting heterogeneous precipitation of elemental Al. Owing to aluminum’s exceptionally high affinity for oxygen, this precipitated Al rapidly oxidizes upon exposure to trace oxygen [19], yielding thermodynamically stable Al_2_O_3_ [21]. The nucleation and growth of Al_2_O_3_ deplete local oxygen activity, establishing a sustained chemical potential gradient that continuously drives additional Al diffusion, precipitation, and oxidation. This autocatalytic cycle culminates in localized clustering of brittle Al_2_O_3_ particles, which act as pore nucleation sites and impede densification—leading to a measurable increase in both average pore size and pore number density, as shown in Figure 3g,h. Table 4 summarizes the EDS point analysis results for positions A1–A6 in Figure 3h. It shows that Al is predominantly enriched in the binder phase; this discovery is consistent with the results of the previous XRD analysis. In contrast, EDS point analysis of the dark-contrast pore regions (A6) reveals intense Al enrichment, with local Al concentrations reaching up to 29.6 wt.%, strongly suggesting that these features correspond to embedded Al-rich oxide precipitates (e.g., Al_2_O_3_ or Al–Co–O phases). It should be noted that micropore formation or the precipitation of brittle Al_2_O_3_ phases synergistically degrades the material’s mechanical properties.

**Figure 5:**
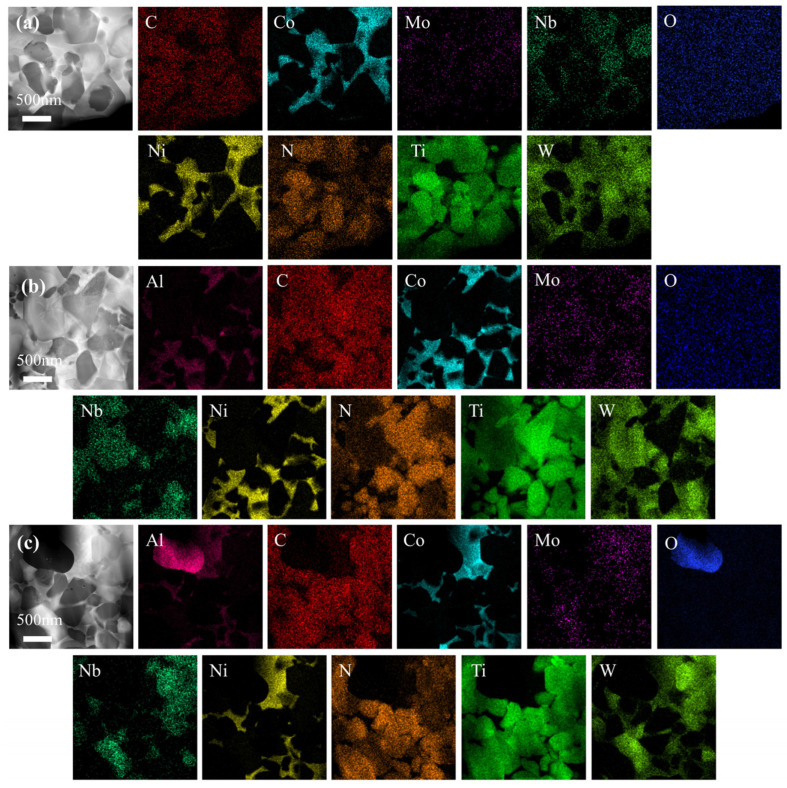
(HAADF) STEM images of samples A0, A1, and A3, along with their corresponding EDS element mappings: (**a**) A0, (**b**) A1, and (**c**) A3.

To systematically elucidate the microstructural characteristics and element distribution behavior of the investigated cermets, TEM and HRTEM analyses were performed on the samples A0, A1, and A3, complemented by EDS mapping. Figure 5 displays the EDS mapping images of all three samples, illustrating the spatial distribution of key constituent elements—including Ti, W, Nb, Mo, Co, Ni, Al, C, N, and O—across the A0, A1, and A3 specimens. Compared with sample A0, in sample A1, which contained 1.0 wt.% AlN, aluminum was detected. The distribution of Al elements was highly consistent with that of cobalt and nickel elements. No aluminum was found in other areas, and no significant oxygen was detected in either sample. This observation is consistent with the XRD and SEM findings, confirming that Al liberated from AlN decomposition fully dissolved into and homogeneously distributed throughout the Co/Ni binder phase. However, when the AlN content was increased to 3.0 wt.%, distinct aluminum-rich agglomerates formed in the A3 sample, and oxygen was exclusively enriched within these agglomerates—providing direct metallographic evidence for Al_2_O_3_.

**Figure 6:**
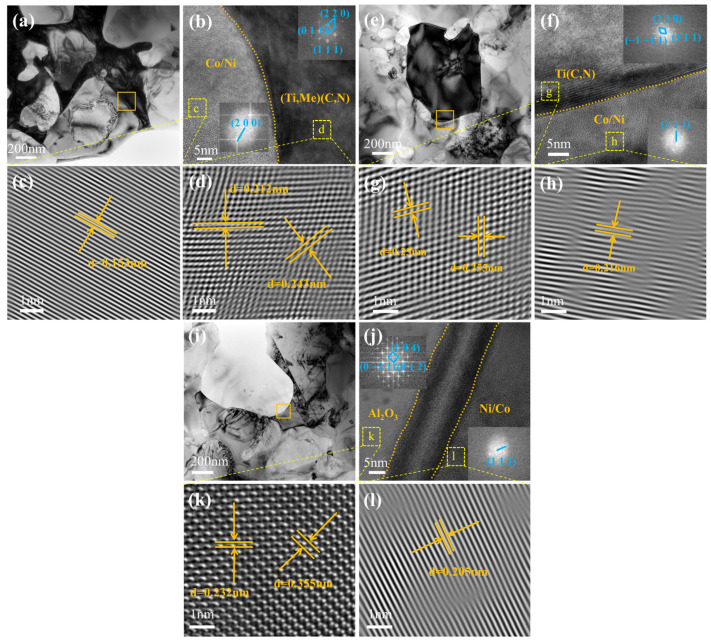
TEM and HRTEM images of the A0 (**a**–**d**), A1 (**e**–**h**), and A3 (**i**–**l**). (**a**,**e**,**i**) Low-magnification TEM images; (**b**,**f**,**j**) HRTEM images showing the lattice fringes of the regions boxed in (**a**), (**e**), and (**i**), respectively; (**c**,**d**), (**g**,**h**), and (**k**,**l**) are the corresponding FFT patterns of the regions shown in (**b**), (**f**), and (**j**), respectively.

Figure 6 shows the TEM and HRTEM images of the samples. The interfacial analysis in Figure 6a–d indicates strong interfacial bonding between the gray rim phase and the binder phase in the A0 cermet. Figure 6e–h shows the semi-coherent interface between the rimless black core and the binder phase in sample A1. It can be observed that the addition of AlN increases the lattice fringe intensity of the binder phase, making its strength more compatible with that of the hard phases. This demonstrates favorable interfacial bonding between the core and the binder phase. In contrast, Figure 6i–l reveal the interfacial features between Al_2_O_3_ and the binder phase in sample A3. Due to the significant difference in interplanar spacing between Al_2_O_3_ and the binder phase, a severe lattice mismatch occurs. This lattice mismatch consequently leads to an indistinct interface and poor interfacial bonding strength, which further leads to the deterioration of the mechanical properties of the material.

### 3.3. Mechanical Performance

**Figure 7:**
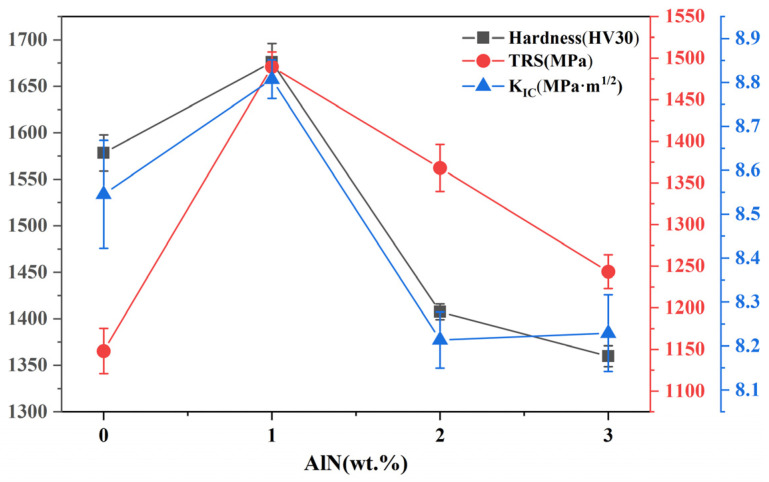
Mechanical performance (hardness (HV30), fracture toughness (K_IC_), together with trans verse rupture strength (TRS) of cermets.

Vickers hardness (HV30), fracture toughness (K_IC_), and three-point bending tests were conducted to evaluate the mechanical properties of the sintered Ti(C,N)-based cermets at room temperature. As shown in Figure 7, hardness, fracture toughness, and flexural strength exhibit a systematic variation with increasing AlN content. The results indicate that an optimal AlN addition (1.0 wt.%) markedly enhances the overall mechanical performance: sample A1 achieves peak values in all three properties—1676 HV30, 8.8 MPa·m^1/2^, and 1486 MPa, respectively. Such a significant improvement in performance is attributed to the addition of AlN, which simultaneously inhibits grain growth and strengthens the binder phase. This is directly supported by multiple lines of evidence, including SEM grain size statistics (the average grain size decreased from 0.75 μm to 0.69 μm), XRD lattice expansion, and TEM observations revealing enhanced interfacial bonding strength. However, further increasing the AlN content leads to a decline in material performance: at 2.0 wt.%, micropores emerge in the microstructure, causing significant reductions in hardness and fracture toughness; at 3.0 wt.%, pores induced by Al_2_O_3_ agglomeration are additionally observed, resulting in further deterioration of comprehensive mechanical performance. As shown in Table 5, the cermets’ density exhibits a trend consistent with their comprehensive mechanical properties: it peaks at 1.0 wt.% AlN addition and then declines with further increases in AlN content. Based on the comprehensive evaluation of mechanical properties and microstructural evolution, the critical threshold for AlN addition lies between 1.0 wt.% and 2.0 wt.%: below this threshold, beneficial effects dominate; above it, detrimental effects (agglomeration and porosity) begin to outweigh the beneficial effects, resulting in a gradual decline in performance.

**Figure 8:**
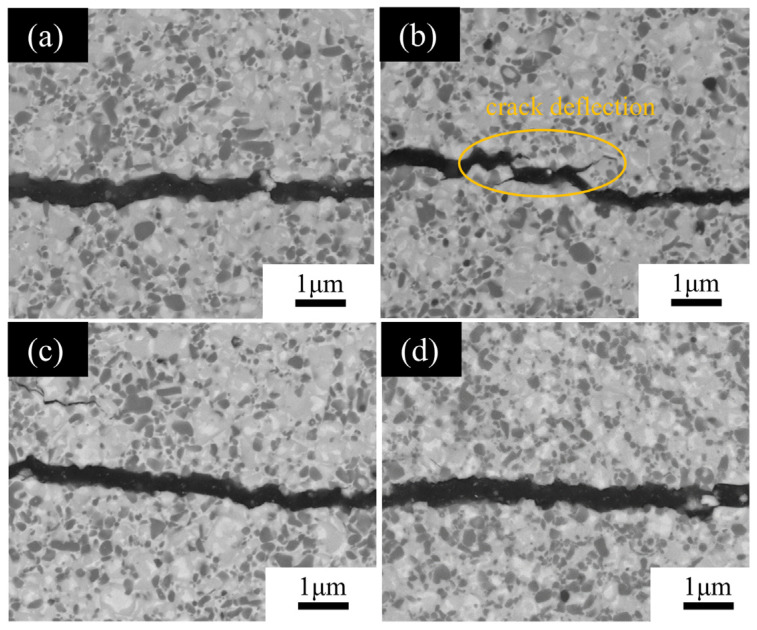
Crack propagation images of Ti(C,N)-based cermets with different AlN contents: (**a**) A0, (**b**) A1, (**c**) A2, and (**d**) A3.

The fracture mechanisms of the samples were examined by analyzing their crack propagation characteristics. Ti(C,N)-based cermets were evaluated for fracture surface features across the AlN compositions. As illustrated in Figure 8, the cracks display fully developed fracture paths, propagating predominantly through intergranular and transgranular modes. In Figure 8a, in the AlN-free cermet, the crack propagates in a continuous manner with a relatively coarse width, accompanied by distinct intergranular fracture characteristics. In contrast, Figure 8b reveals that with the addition of 1.0 wt.% AlN, the crack path significantly deflects and becomes notably finer, whereas the fracture surface exhibits typical transgranular features. When the AlN addition exceeds 2.0 wt.%, the crack width becomes progressively larger, whereas the fracture toughness decreases with increasing AlN content. This behavior is attributed to the micropores in the microstructure of the sample A2. When the AlN content was increased to 3.0 wt.%, the number and size of micropores in the microstructure increased further due to the formation of Al_2_O_3_ aggregates; consequently, the widening of intergranular cracks reflects the deterioration of mechanical performance induced by these internal defects.

**Figure 9:**
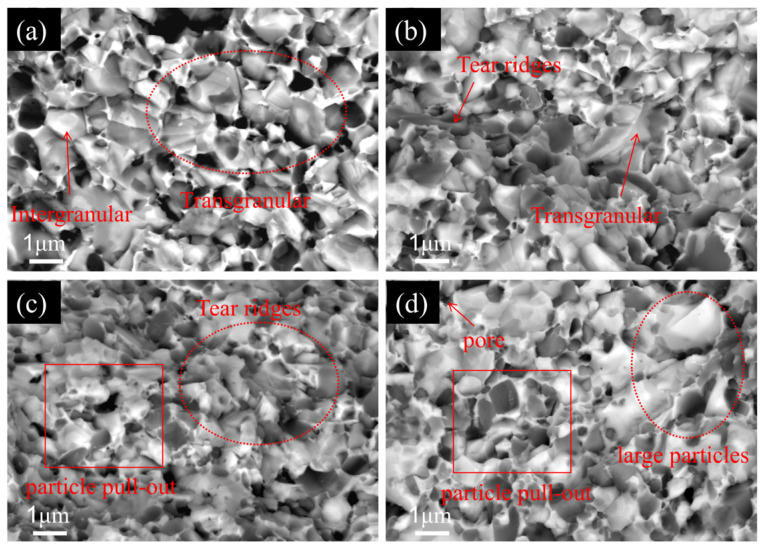
Fracture morphologies of Ti(C,N)-based cermets with different AlN addition amounts: (**a**) A0, (**b**) A1, (**c**) A2, and (**d**) A3.

Figure 9 presents SEM fractographs of Ti(C,N)-based cermets with different AlN addition levels. In Figure 9a, pits left by the pull-out of hard phase particles are observed on the fracture surface, indicating intergranular fracture of some grains; meanwhile, river-like cleavage patterns are visible on the surfaces of Ti(C,N) grains, suggesting that the fracture mode also includes transgranular fracture. At AlN addition levels of 0 wt.% and 1.0 wt.%, transgranular fracture features dominate. Compared with sample A0, sample A1 shows fewer pull-out features and a higher proportion of transgranular fracture, reflecting a strong interfacial bonding strength between the hard phase and the binder phase; consequently, sample A1 exhibits higher flexural strength and toughness. With a further increase in AlN addition, samples A2 and A3 display an increased occurrence of particle pull-out, along with larger pulled-out particles, resulting in extensive exposure of the binder phase. Moreover, micropores appear on the fracture surface of sample A3. Collectively, these factors severely degrade the mechanical properties of the material.

## 4. Conclusions

This study systematically investigates the effect of aluminum nitride (AlN) addition on the microstructure and mechanical properties of Ti(C,N)-based cermets with high nitrogen content and a low-binder-phase fraction. Four distinct compositions were fabricated by precisely controlling the AlN content from 0 to 3.0 wt%. The principal findings are as follows:

(1) Phase and microstructural analyses reveal that, at AlN additions up to 1.0 wt.%, aluminum atoms preferentially dissolve into the Ni/Co binder phase, forming an Al–Ni–Co intermetallic strengthening phase. This dissolution enhances binder-phase strength and suppresses the dissolution–reprecipitation mechanism, thereby effectively refining the Ti(C,N) grains. In contrast, AlN additions of 2.0 wt.% and above promote in situ oxidation during sintering: micropores emerge at 2.0 wt.%, while pronounced Al_2_O_3_ brittle-phase agglomerates form at 3.0 wt.%, both of which significantly impair microstructural homogeneity and integrity.
(2) Relative density, hardness, fracture toughness, and transverse rupture strength collectively exhibit a unimodal dependence on AlN content—peaking at 1.0 wt.% and declining monotonically with further increases—thereby establishing a narrow optimal compositional window for achieving balanced densification and superior mechanical performance.
(3) At 1.0 wt.% AlN, the Ti(C,N)-based cermets achieve optimal mechanical performance, exhibiting a hardness of 1676 HV30, a transverse rupture strength of 1486 MPa, and a fracture toughness of 8.8 MPa·m^1/2^.
(4) The present study finds that in the high-nitrogen, low-binder system, micropore defects already appear when the AlN addition reaches 2.0 wt.%, which is considerably lower than the value (~5.0 wt.%) reported for low-nitrogen, high-binder systems in the literature [12]. This can be attributed to two factors: the limited solubility of Al in the binder phase and the higher nitrogen dissociation tendency of the high-nitrogen starting powder. Further research is warranted to explore the application potential of this Al-strengthened binder phase system in improving the high-temperature performance of cermets.

## Figures and Tables

**Table 1:** Purity, particle size distribution, oxygen content, and supplier of the raw materials.

Name	Purity/wt.%	Particle Size/μm	Oxygen Content/wt.%	Supplier
Ti(C_0.5_,N_0.5_)	99.9	1	0.494	Zhuzhou Haokun Hard Materials Co., Ltd., Zhuzhou, China
WC	99.9	0.5	0.189	Xiamen Golden Egret Special Alloy Co., Ltd., Xiamen, China
Mo_2_C	99.9	0.5	0.341	Zhuzhou Haokun Hard Materials Co., Ltd. Zhuzhou, China
NbC	99.9	0.5	0.368	Zhuzhou Haokun Hard Materials Co., Ltd. Zhuzhou, China
Co	99.9	0.8	-	Shanghai Pantan Powder Materials Co., Ltd., Shanghai, China
Ni	99.9	2	-	Shanghai Pantan Powder Materials Co., Ltd. Shanghai, China
AlN	99.9	0.6	1.023	Shanghai Pantan Powder Materials Co., Ltd. Shanghai, China
(HO(CH_2_CH_2_O)_n_H)	99.9	-	-	Shanghai Macklin Biochemical Co., Ltd., Shanghai, China
DI water	-	-	-	Self-made

**Table 2:** Composition of the as-prepared test specimens (wt.%).

Cermets	Ti(C_0.5_,N_0.5_)	WC	Mo_2_C	NbC	Co	Ni	AlN
A0	51	24	1	8	8.5	7.5	0
A1	51	23	1	8	8.5	7.5	1
A2	51	22	1	8	8.5	7.5	2
A3	51	21	1	8	8.5	7.5	3

**Table 3:** Lattice parameters of the Co/Ni binder phase in the four sintered cermets (calculated according to their XRD patterns).

Sample	A0	A1	A2	A3
Lattice parameter (Å)	3.596 ± 0.0006	3.600 ± 0.0005	3.601 ± 0.0006	3.597 ± 0.0007

**Table 4:** EDS point scanning results of points A1 to A_6_ in Figure 3h.

Element (Wt.%)	A_1_ Black Core	A_2_ Gray Rim Surrounding Black Core	A_3_ White Core	A_4_ Gray Rim Surrounding White Core	A_5_ Binder Phase	A_6_ Dark Pore
Co	5.39	1.31	0.8	0.72	17.09	2.26
Ni	3.2	1.16	0.49	0.44	18.47	2.07
Al	0.47	0.48	0.82	0.72	2.19	29.6
W	5.18	24.36	33.73	30.07	17.39	8.71
Ti	65.9	46.48	33.74	39.97	26.12	16.02
Mo	0.23	1.21	2.46	0.73	0.88	0.75
Nb	0.81	10.44	15.73	14.71	4.23	2.78
C	10.3	10.64	11.92	11.65	7.77	7.88
N	7.44	1.83	0.31	0.74	5.3	1.6

**Table 5:** Actual density, theoretical density, and relative density.

Cermets	Theoretical Density (g/cm^3^)	Actual Density (g/cm^3^)	Relative Density/%
A0	7.0068	6.6310	94.7
A1	6.8949	6.6415	96.3
A2	6.7666	6.4805	95.8
A3	6.6717	6.2980	94.4

## Data Availability

The original contributions presented in this study are included in the article. Further inquiries can be directed to the corresponding author.
